# Implementing a real-time, AI-based, people detection and social distancing measuring system for Covid-19

**DOI:** 10.1007/s11554-021-01070-6

**Published:** 2021-01-21

**Authors:** Sergio Saponara, Abdussalam Elhanashi, Alessio Gagliardi

**Affiliations:** grid.5395.a 0000 0004 1757 3729 Dip. Ingegneria Dell’Informazione University of Pisa, Via G. Caruso 16, 56122 Pisa, Italy

**Keywords:** COVID-19, Neural network, Social distancing, Temperature analysis, Jetson nano, Distributed surveillance system

## Abstract

COVID-19 is a disease caused by a severe respiratory syndrome coronavirus. It was identified in December 2019 in Wuhan, China. It has resulted in an ongoing pandemic that caused infected cases including many deaths. Coronavirus is primarily spread between people during close contact. Motivating to this notion, this research proposes an artificial intelligence system for social distancing classification of persons using thermal images. By exploiting YOLOv2 (you look at once) approach, a novel deep learning detection technique is developed for detecting and tracking people in indoor and outdoor scenarios. An algorithm is also implemented for measuring and classifying the distance between persons and to automatically check if social distancing rules are respected or not. Hence, this work aims at minimizing the spread of the COVID-19 virus by evaluating if and how persons comply with social distancing rules. The proposed approach is applied to images acquired through thermal cameras, to establish a complete AI system for people tracking, social distancing classification, and body temperature monitoring. The training phase is done with two datasets captured from different thermal cameras. Ground Truth Labeler app is used for labeling the persons in the images. The proposed technique has been deployed in a low-cost embedded system (*Jetson Nano*) which is composed of a fixed camera. The proposed approach is implemented in a distributed surveillance video system to visualize people from several cameras in one centralized monitoring system. The achieved results show that the proposed method is suitable to set up a surveillance system in smart cities for people detection, social distancing classification, and body temperature analysis.

## Introduction

COVID-19 is a disease caused by a new coronavirus which appeared in China in December 2019. COVID-19 symptoms include mainly fever, cough, chills, and shortness of breath, body aches, loss of taste, and smell. COVID-19 can be severe, and in many cases, it has caused death. The coronavirus can spread from one person to another as diagnosed by researchers in laboratories. This pandemic has spread to over 188 countries around the world [1]. On October 15, 2020, WHO (World Health Organization) declared that there have been 38,394,169 confirmed COVID-19 cases and 1089,047 deaths [2] around the world. The uncertainty, underpinning, and complexity of the coronavirus have made it difficult to predict the duration and spread of this pandemic. As of yet, there is no vaccine available. Prevention involves wearing masks and washing hands frequently. An infected person should stay at home when people are sick to prevent spreading this pandemic to the others. This situation forces the global community and governments to find the best mitigation plan to stop the spread of coronavirus. Nations stopped their business and closed the border and public places such as schools and workplaces to avoid people’s interactions. It has been reported that all infected countries who applied the lock-down for their communities achieved a reduction of the number of COVID-19 cases and the number of deaths from this pandemic.

Fever or chills are common symptoms of coronavirus. Researchers in China found that 99% of people infected with the coronavirus presented with a high temperature. Thermal cameras and non-contact infrared thermometers, which are non-contact instruments, can be used to measure body temperature. This approach can monitor a person’s surface temperature to limit the spread of coronavirus infections.

Based on the information from the World Health Organization, social distancing is the best practice where individuals can minimize physical contact with possible COVID-19 carriers by maintaining a certain distance between one person and another. The main target is to provide a comprehensive tool and effective technologies that can be utilized to enforce social distancing. Technologies could play an important role to facilitate social distancing practice. In such a context, Artificial Intelligence (AI) and information and communication technology (ICT) can be used in addressing this challenge.

This research aims at mitigating the spread of this virus in communities and saving the lives of people. In this work, we propose a deep learning object detection model for people detection in combination with an implemented algorithm for social distancing classification on thermal images. Hereafter, the paper is organized as follows: after the introduction of COVID-19 in Sects. 1, 2 presents the research background and related work. Section 3 shows an overview of object detection. Section 4 presents the proposed methodology to define a measuring system for people detection and social distancing check. Section 5 shows the experimental results and Sect. 6 describes the implementation of the proposed approach on embedded hardware. Conclusions are drawn in Sect. 7.

## Research background and related work

Social distancing and temperature screening are effective tools for preventing the spread of disease. They have been suggested by many organizations, including the World Health Organization (WHO) [3]. Russel et al. [4] studied the effects of social distancing techniques on the spread of coronavirus. This paper presented scientific location contact patterns to produce the trajectory of an outbreak by utilizing susceptible exposed infected removed (SEIR) methods. The authors also mentioned that the sudden lifting of social distancing could increase the infection and spread of the virus between people. Nabil Kahale [5] highlighted the impact of social distancing measures. The study aimed to derive an approximation that shows how early social distancing measures can reduce economic loss and the number of new infections significantly. At the time when coronavirus is begun spreading across the individuals and society, research and scientists are starting to find out the best solution to eliminate the spread of this pandemic [6, 7]. Jennifer Berglund [8], suggested tracking a person infected with COVID-19 using GPS and built-in applications in smartphones. However, this technology has limitations on tracking individuals who have no Wi-Fi or cell signals. On the other hand, some authorities utilize drones with mounted video cameras to track the gathering of individuals in the outdoor area [9, 10]. Such technology is suitable for monitoring COVID-19 which could amid the coronavirus outbreak.

Recently, the problem of classifying and detecting the objects in an image is solved, thanks to the improvements in computer vision and deep learning in general. Accordingly, computer vision development has focused on various interesting and challenging topics, such as neural style transfer, segmentation, and tracking, and of course object detection [11].

Deep learning is an artificial intelligence function (AI) that emulates the tasks of the human brain in data processing and object detection. It can be referred to as a neural network with a sophisticated algorithm. The history of the neural network dates to 1940 [12]. The original intention of the neural network is to solve learning problems ethically [13]. A convolutional neural network (CNN) is widely used in deep learning models for object detection. CNN is a deep learning algorithm that takes an input image and assigns the learnable weights and biases for various classes in an image and differentiates them from one to another. The convolutional neural network has been made evolution which can be implemented on an embedded system with a low-resolution input and low complexity [14]. There are various deep learning models such as R-CNN, Single-shot detector (SSD), and YOLO which are applied in different applications for object detection. These models are efficient algorithms for movement estimation in video scenes. Ebrahim et al. [15], proposed a technical approach for detecting people using video frames. The author utilized a background subtraction and Gaussian mixture with a deep learning detector for people detection. In method [16], the authors presented a deep learning (CNN) technique for human detection. They utilized a combination of deep learning and machine learning methods to achieve high accuracy and less computation for people detection. Unfortunately, this method had problems with low speed for real-time detection. In method [17], researchers suggested a method on static crowds for a group of people that stayed in the same location for a long time. They utilized the mean of class as support vector machine (SVM) to categorize patches as essential crowds and these patches are extracted by text features.

Recent developments showed that the identification of individuals through video surveillance cameras can be achieved by face [18], and a person’s manner of walking. However, the detection of a person under crowds’ technique is difficult and hard to optimize.

In method [19], the authors presented a solution for detecting pedestrians with a low-resolution camera by utilizing background subtraction by extracting foreground silhouettes and classifying them in real-time.

## Overview of object detection

**Fig. 1:**
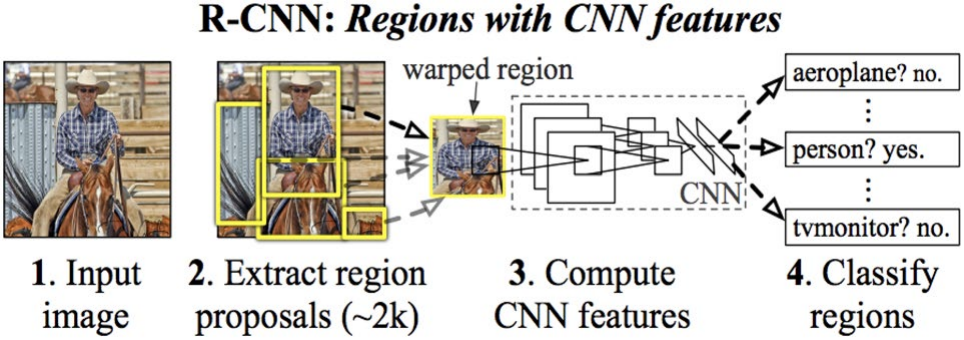
The schematic diagram for R-CNN detector

Object detection systems place a bounding box around the objects and associate the correct object’s category with each bounding box. Deep learning is an effective method to perform object detection. In [20], Ross Girshick explored a regional convolutional neural network detector (R-CNN). This model consists of four stages. It starts with introducing the images into the input layer, then it extracts the regional proposals, after that it computes the features by CNN, and finally, it classifies these features, see Fig. 1. R-CNN uses selective search algorithms to generate region proposals. It takes a huge amount of time as it would have to classify the regions per image. R-CNN cannot be implemented in real-time object detection as it takes 47 s for each image. R-CNN cannot be trained at one time. Rather, it needs to train every part independently.

**Fig. 2:**
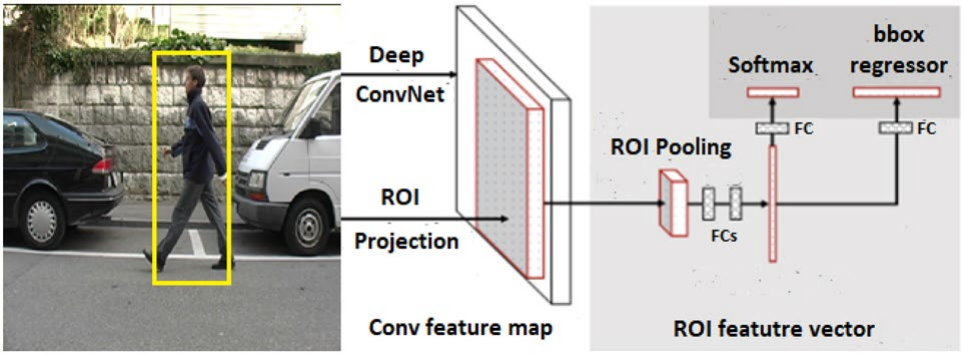
The schematic diagram for Fast R-CNN detector

Fast R-CNN is another version of the regional proposal algorithm, which was presented by the same author of R-CNN model [21]. Fast R-CNN enhanced the drawbacks from R-CNN to build faster object detection algorithm. It is similar to the R-CNN algorithm. However, the input image is fed to the convolutional neural network to generate a convolutional feature map instead of feeding region proposals to CNN. The region proposal is warped into squares in this model. Using region of interest (ROI) pooling layer, these regions are reshaped into a fixed size which can be fed into a fully connected layer. Softmax layer is used in this architecture to predict the class of region and the offset values of the bounding box. Figure 2 shows the schematic diagram for fast R-CNN detector.

Both algorithms (R-CNN and Fast R-CNN) use selective search to find the region proposals. This process a slow and time-consuming which is affecting the performance for neural network algorithm.

Recent improvements in object detection deep learning include other algorithms such as YOLO and YOLOv2. You look at one or (YOLO) is a state-of-the-art deep learning object detection. It was presented by Joseph Redmon et al. [22]. YOLO uses a single neural network to the whole image. It divides the image into regions and predicts the bounding boxes and the probabilities for each region. These bounding boxes are weighted by predicted probabilities.

**Fig. 3:**
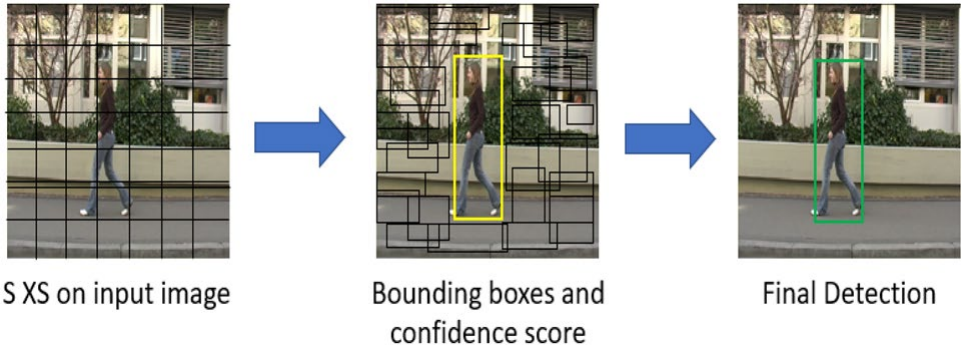
Schematic diagram for YOLO: input image which splits into S×S grids, each grid predicts the bounding boxes and the confidence scores and finally, the score encodes the probability with bounding box on the detected class

YOLO detector looks full image at one time; therefore, its predictions are informed by the context in the image. It predicts with single network evaluation, unlike other object detectors such as (R-CNN) which requires thousands for a single image. YOLO algorithms take the input image and split into *S ×S* grids. It extracts the features from each grid. It predicts the bounding boxes with confidence scores for the predicted classes in the bounding boxes, see Fig. 3. Each grid cell detects bounding boxes and confidence scores. The bounding box consists of five predictions which are represented with (*x*, *y*, *w*, *h*) and the confidence score. The (*x*, *y*) coordinates reflect the center of the bounding box of the grid cell. The (*w*, *h*) represents the width and the height of the full image. The confidence scores represent the measurement of how confident the detector is that the box contains the object to be predicted.

YOLO predicts several bounding boxes for each grid cell. In the training stage, it only requires one predictor of the bounding box to be responsible for each class. The predictor is assigned to predict an object which has the highest Intersection over Union value (IoU) for the ground truth. This process leads to specialization within the bounding boxes prediction. YOLO algorithms use sum-squared error between the ground truth and predictions of bounding boxes for loss. This sum squared error computes the classification, localization, and confidence losses for the model. Therefore, YOLO is optimized with the following loss function to enhance its performance during the training process, see Eq. (1). YOLOv2 is the second version of YOLO. It is an object detection system targeted for real-time processing. It has several improvements to YOLO as explored in YOLO9000 paper [23]. YOLOv2 resolved the issues which were encountered with YOLO, thus improving the processing accuracy and speed for the architecture. It enhanced the errors of localization for the classes to be predicted in the images. It uses batch normalization in all convolutional layers.

1 $$ \begin{aligned}&{\lambda }_{\mathrm{coord}}\sum_{i=0}^{{s}^{2}}\sum_{j=0}^{B}{1}_{i,j}^{\mathrm{obj}}[({x}_{i}-{\stackrel{\wedge }{x}}_{i}{)}^{2}+\left({y}_{i}-{\stackrel{\wedge }{y}}_{i}{)}^{2}\right]\\ &\quad+\hspace{0.33em}{\lambda }_{\mathrm{coord}}\sum_{i=0}^{{s}^{2}}\sum_{j=0}^{B}{1}_{i,j}^{\mathrm{obj}}[({\sqrt{w}}_{i}-{{\sqrt{\stackrel{\wedge }{w}}}_{i})}^{2}+\left({\sqrt{h}}_{i}-{\sqrt{\stackrel{\wedge }{h}}}_{i}{)}^{2}\right]\\ &\quad+\hspace{0.33em}\sum_{i=0}^{{s}^{2}}\sum_{j=0}^{B}{1}_{i,j}^{\mathrm{obj}}({C}_{i}-{\stackrel{\wedge }{C}}_{i}{)}^{2} +\hspace{0.33em}{\lambda }_{\mathrm{noobj}}\sum_{i=0}^{{s}^{2}}\sum_{j=0}^{B}{1}_{i,j}^{\mathrm{noobj}}({C}_{i}-{\stackrel{\wedge }{C}}_{i}{)}^{2}\\ &\quad +\sum_{i=0}^{{s}^{2}}{1}_{i}^{\mathrm{obj}}\sum_{c\in \mathrm{class}}^{ }({p}_{i}\left(c\right)-{\stackrel{\wedge }{p}}_{i}\left(c\right){)}^{2},\end{aligned} $$

where: ${\lambda }_{\mathrm{coord}}$ is a constant used to increase the weight for the first two terms of the loss function. $B$ is the number of box predictions for each cell. *s*^2^ is the number of cells. ${1}_{i,j}^{\mathrm{obj}}$ is equal to 1 if there is an object in cell *i *and confidence of the *j*th predictor of this cell is the highest among all the predictors of this cell. ${x}_{i}$,${ y}_{i}$ represent the location of centroid of the anchor box. ${w}_{i}$ is the width of the anchor box. ${h}_{i}$ is the height of the anchor box. ${C}_{i}$ is the confidence score whether there is an object or no. ${\stackrel{\wedge }{C}}_{i}$ is the box confidence score of the box $j$ in cell $i$. ${\lambda }_{\mathrm{noobj}}$ weights down the loss when detecting background. ${1}_{i,j}^{\mathrm{noobj}}$ is the complement of ${1}_{ij}^{\mathrm{obj}}$. ${1}_{i}^{\mathrm{obj}}$ = 1 if an object appears in the cell $i$, otherwise 0. ${p}_{i}(c)$ is the classification loss. ${\stackrel{\wedge }{p}}_{i}(c)$ is the conditional class probability for class $c$ in cell $i$.

Batch normalization helps the regularization of the model. It eliminated the requirement for using the dropout layers to overcome the overfitting problems. It improves the normalization for its input by defining the variance values and means over the mini-batch and it calculates the activation as seen in Eq. (2)

2 $${\widehat{x}}_{i}=\frac{xi-{\mu }_{B}}{\sqrt{ {\sigma }_{B}^{2}+\epsilon }}, $$

where ${\widehat{x}}_{i}$ is the normalize value. $xi$ is the element of the input. ${\mu }_{B}$ is the mini-batch mean. ${\sigma }_{B}^{2}$ is the batch variance. $\in$  is the property of Epsilon and enhances the mini batch when the variance value is small.

We used anchor boxes to make bounding boxes on the detected objects in the images. These boxes are a set of predefined rectangular boxes with a specific width and height. Anchor boxes are defined to capture the scale and ratio of certain classes that are to be detected and typically selected based on the sizes of the objects in the training dataset. K-mean clustering was used to select a good set of labeled boxes in the training dataset in MATLAB. It is essential to have the correct sizes of these bounding boxes (height, width) for YOLOv2 to detect the targeted objects accurately. Intersection over Union (IoU) score of k-means was measured to determine the required number of these bounding boxes for the detector. The advantage of using anchor boxes is to prevent utilizing more boxes which could lead to overfitting and poor performance for YOLOv2 model.

## Proposed methodology

### Social distancing detector steps

**Fig. 4:**
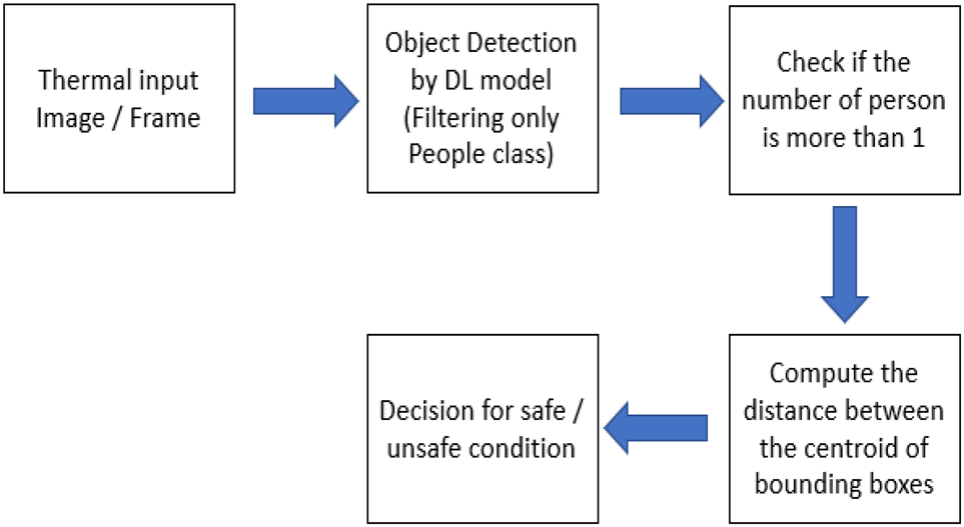
The steps involved for people detection and social distancing classification on thermal images

This section discusses the essential steps which are attempted to establish a workflow for monitoring social distancing on thermal images as seen in Fig. 4:

1. Prepare the thermal images or streaming a video from a thermal camera which contains people.
2. Applying the deep learning object detector to detect people in thermal images or video streams.
3. Check the number of persons that are in the images or video stream.
4. Compute the distance between the centroid of the bounding boxes which are enclosed to the detected people.
5. Finally, the algorithm will decide for safe or unsafe social distancing based on the number of persons and the measured distance between the centroid of bounding boxes.

### Neural network design

**Fig. 5:**
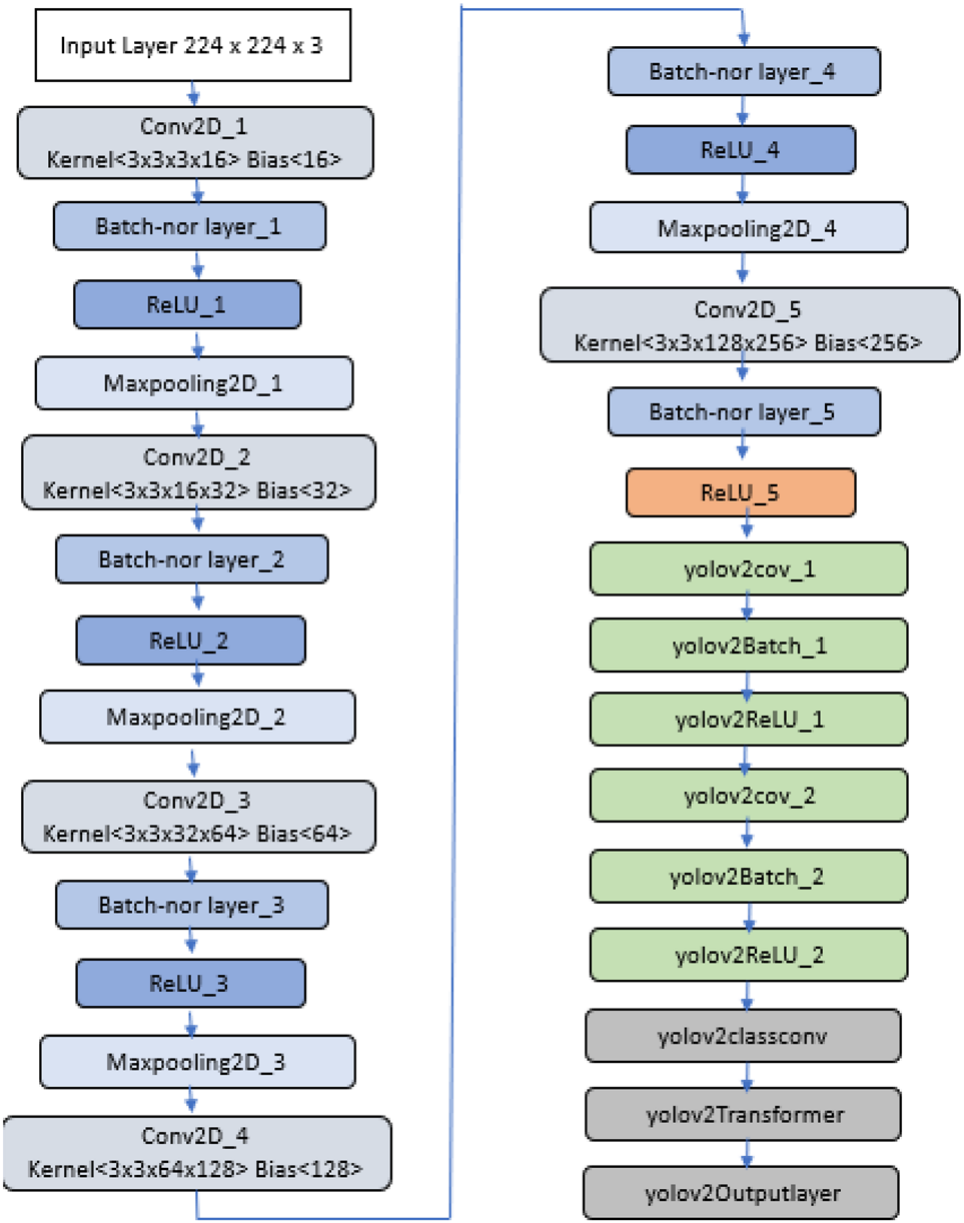
Architecture of YOLOv2 Neural Network

A Deep Neural Network (DNN) application is used in MATLAB to construct YOLOv2 neural network layers. Then the designed DNN is ported in embedded platforms like NVIDIA Jetson Nano. We built a CNN with 29 layers, see Fig. 5. This is to establish a light-weight model to fit the real-time implementation of CNN inference also in low-cost embedded platforms, such as those of IoT nodes. The neural network layers include the input layer, middle layers, and subnetwork of YOLOv2 layers.

The proposed approach starts with the input image layer, which introduces the input image with a size of (224 × 224 × 3) for our detector. A set of middle layers was used, which includes batch normalization, convolutional, max-pooling, and Relu (rectified linear unit) layers. Convolutional layers were used to map the features for the images. The size of the filter was set to (3 × 3). It defines the height and width of the regions in the input image. Batch normalization layers were used to regularize the model and eliminate the overfitting problem. ReLU activation functions were utilized to introduce the non-linearity to the neural network. Maxpooling layers were used to downsample the images into pooling regions. We applied (2 × 2) for the size of pooling with a stride of (2 × 2) for all max-pooling layers in a neural network. ‘ReLU_5’ was used as the feature extraction layer. This is to extract the features from neural network layers and then given as input to YOLOv2 subnetwork layers. YOLOv2 layers were used in this detector which constructs YOLOv2 detection network. YOLOv2 Subnetwork consists of a batch of layers that include convolutional (yolov2cov), batch normalization (yolov2Batch), ReLU (yolov2ReLU), transform, and output layers. The transform layer was utilized in YOLOv2 detector to stabilize the network for object localization. This layer transforms the raw CNN output into a form required to produce object detections. YOLOv2 output layer was used which refines the location of bounding boxes to the detected objects. The model was examined with a neural network analyzer and reported zero errors.

### Training

**Fig. 6:**
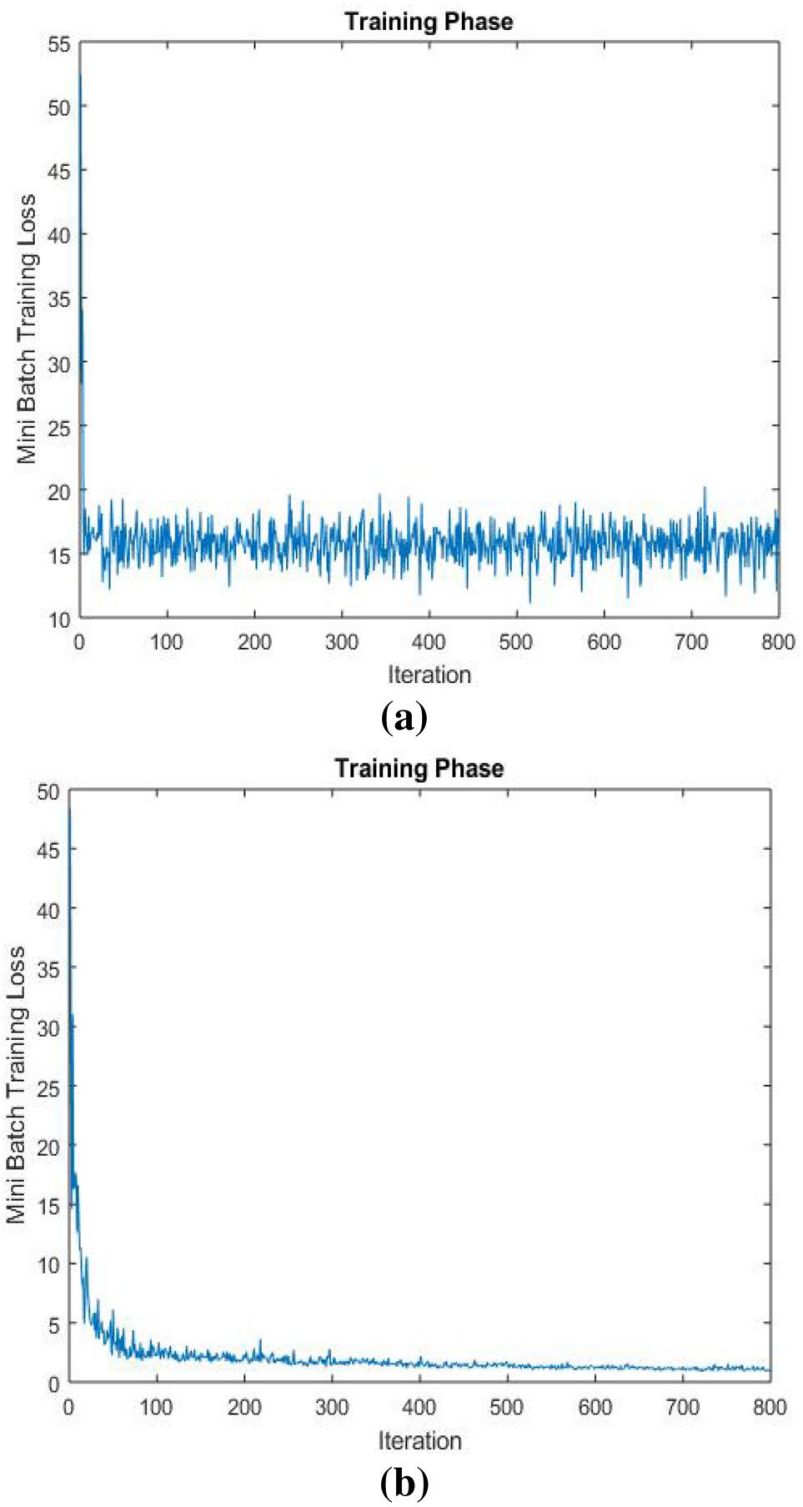
**a** Mini-Batch Loss Curve before fine-tuning, **b** Mini-Batch Loss Curve after fine-tuning

The designed network was trained with two different datasets of thermal images. Dataset I consists of 775 thermal images of humans captured in various scenarios while walking, running, or sneaking and in different body positions, as well as different motion speeds, maximizing the simulated conditions for detecting people in the surveillance and monitoring areas. These images were collected from different sources on the internet. Dataset II consists of 800 images. These images are infrared images that were created by FLIR company for thermal cameras [24]. We used ground truth labeler application in MATLAB for labeling the persons in the thermal images [25]. We split the images into 70% for training, 20 for validation, and 10% for testing for each dataset. The model has been trained with stochastic gradient descent (sdgm) [26]. The learning rate parameter in the training option was used to control the model change in response to the error [27]. We started the learning rate with 10^–2^. However, we noticed that the model was unstable during the training process. The learning rate was fine-tuned at 10^–3^, and the loss curve for mini-batch was steady with small fluctuation, see Fig. 6. Table 1 shows Training Hyper-Parameters for the proposed neural network.

**Table 1:** Training Hyper-Parameters for the proposed neural network

Parameter	Method
Training options	sdgm
L2 Regularization	0.06
Number of epochs	80
Verbose Frequency	50
Mini-batch size	16
Learning rate	0.001

### Algorithm for distancing classification

We also implemented code in MATLAB to work with bounding boxes of a detected person in the thermal images. This code classifies and decides if persons in the image are within safe distancing or not. We assigned a green color for safe social distancing and red color for unsafe social distancing for the bounding boxes. First, we find the number of persons in the images. If it is one person, a green color is assigned for a bounding box of detected persons. When we have two or more persons, then color is decided from the function which is called find Color. This function will determine if the bounding box is 2 or more and in addition to that, it will calculate the distance between the centers of bounding boxes for the detected person. The center points, *C* (*x, y)* of bounding boxes is measured using the equation as seen in Eq. (3).

3 $$ C(X,Y) = \frac{{X_{\min } + X_{\max } }}{2},\frac{{Y_{\min } + Y_{\max } }}{2}, $$

where: $C$ is the center point of the bounding box. *X*_min_ and *X*_max_ are the minimum and maximum values for the corresponding width of the bounding box. *Y*_min_ and *Y*_max_ are the minimum and maximum values for the corresponding height of the bounding box.

**Fig. 7:**
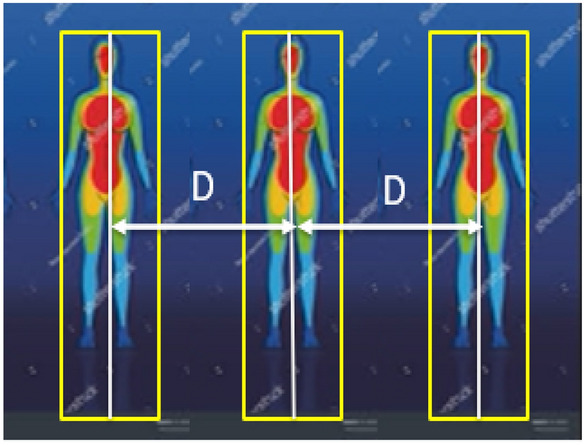
The measured distance (*D*) between the center of each bounding box for a detected person

To measure the distance *C*_1_ (*X*_max_–*X*_min_), and *C*_2_ (*Y*_max_–*Y*_min_), between the center of each bounding box, we used the Euclidean formula, see Eq. (4), where the distance between pixels is translated in a metric distance (knowing the range and field of view covered by the camera) and then compared to a threshold value. In case of finding- color function detects two bounding boxes and the distance is less than the threshold value, these boxes will have a red color. If this function detects two bounding boxes and the distance is more than the threshold value, the color will be green for these boxes. Figure 7 provides the measured distance (D) between the center of each bounding box for a detected person.

4 $$ D(C_{1} ,C_{2} ) = \sqrt {(X_{\max } - X_{\min } )^{2} + (Y_{\max } - Y_{\min } )^{2} ,} $$

where: *D* is the distance between the centers of bounding boxes.

Closed-circuit television (CCTV) cameras are installed in such a way that they provide angle views on the ground plan. To calculate the distance between the people effectively, a top view of the ground plane is required. This can be performed by applying a homography transformation to the four points coordinates in the angled view. These four points can be transformed as shown in the Eq. (5).

5 $$ \left[ {\begin{array}{*{20}c} {X_{{{\text{corn.top}}}} } \\ {Y_{{{\text{corn.top}}}} } \\ 1 \\ {} \\ \end{array} } \right] = M*\left[ {\begin{array}{*{20}c} {X_{{{\text{corn.ang}}}} } \\ {Y_{{{\text{corn.top}}}} } \\ 1 \\ \end{array} } \right], $$

where: *X*_corn.ang_ and *Y*_corn.ang_ represent the pixel coordinates of one of the four points in the CCTV view image *X*_corn.top_, *Y*_corn.top_ represent the same point after transformed to the top view. $M$ is the homography matrix.

To estimate the distance between people in the real world, the distance is calculated between the individuals using Eq. 4 and four points coordinates with homography matrix value. This distance is then scaled by factor $S$ to have the real-world distance between the individuals. The scaling factor $S$ is obtained by measuring a number of pixels in an image that represents 1 m in the real-world.

## Experimental results

**Fig. 8:**
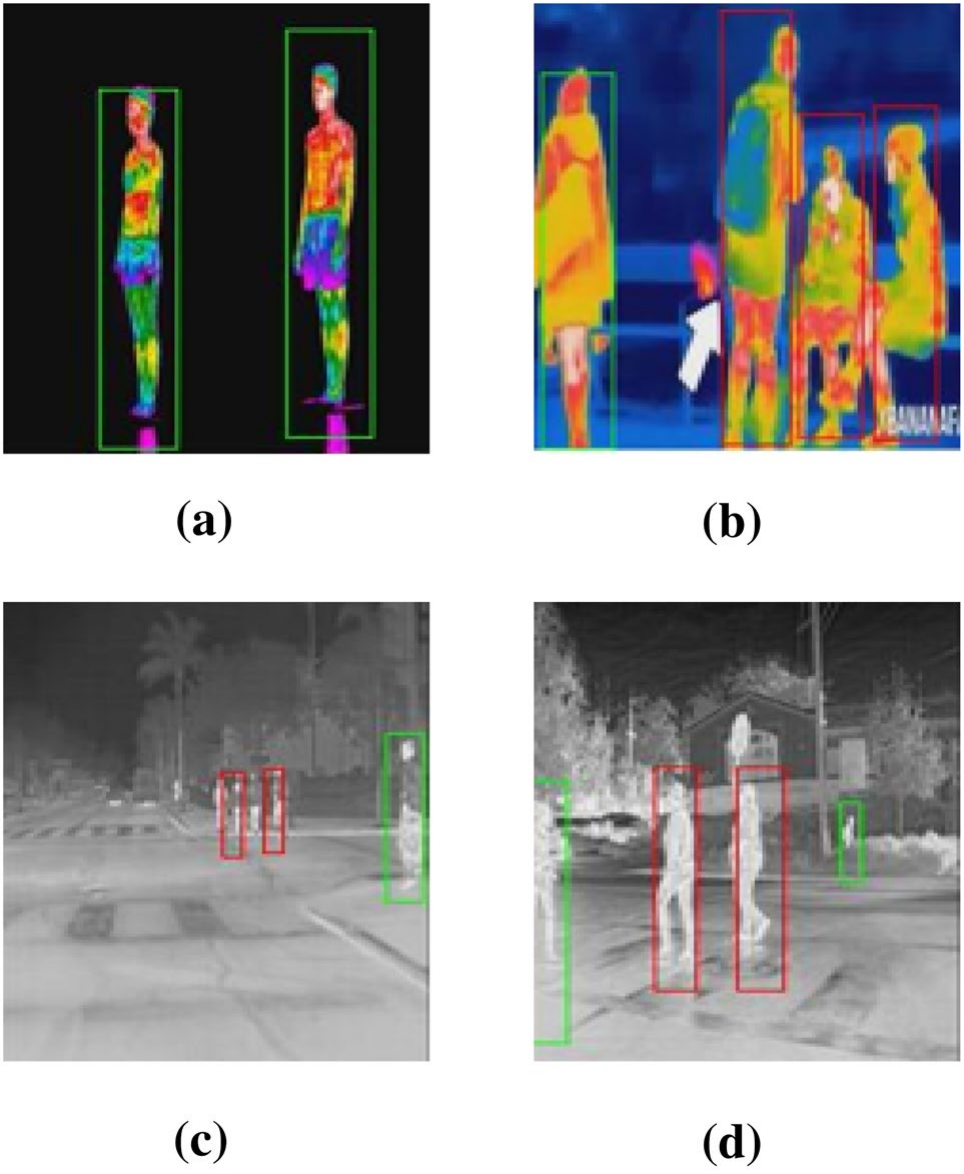
Sample Images from **a**, **b** Dataset I, **c**, **d** Dataset II

The technique proposed in Sect. 4 was examined with two testing datasets to evaluate the capability of detection and localization of persons in the thermal images. These datasets have been made challenging, which encountered a realistic situation by capturing body temperature on people from real thermal cameras. Motivating to that, we selected these datasets for our experiments. YOLOv2 and distance classification algorithms were applied to these thermal images. YOLOv2 model detects people and provides the bounding box information. After people detection, the Euclidean distance between each detected centroid pair is computed using the detected bounding box and its centroid information based on dimensions of (*x*, *y*) for each bounding box. As a further step, we designed and trained R-CNN and Fast R-CNN models for people detection with the same training datasets. We compared these R-CNN and Fast R-CNN architectures with the technique proposed in Sect. 4 using the same testing datasets of thermal images. To measure the efficiency of the proposed approach, the parameters on which the three architectures are evaluated include accuracy, precision, and recall values using confusion matrix criteria, see Eq. (6). Based on the results from these experiments, the new proposed detector showed good performance for people detection, social distancing classification on thermal images in both datasets, see Fig. 8. It achieved significant results with two datasets and overcomes R-CNN and Fast R-CNN detectors see Table 2. YOLOv2 neural network looks the entire image at one time, unlike R-CNN and Fast R-CNN methods which see only the generated region proposals. Therefore, the proposed technique reduces the problem of background mistakes and improves the localization of detected persons in the image. In addition to that, the proposed approach shows better accuracy in comparison to other methodologies [28, 29], and [30], see Table 3. According to these results, the methodology proposed in Sect. 4 is a promising one for people detection and social distancing classification on thermal images.

6 $$ \begin{gathered} {\text{Accuracy = }}\frac{{\text{TP + TN}}}{{\text{TP + FN + TN + FP}}}{,} \hfill \\ {\text{Precision = }}\frac{{{\text{TP}}}}{{\text{TP + FP}}}{,} \hfill \\ {\text{Recall = }}\frac{{{\text{TP}}}}{{\text{TP + FN}}}{,} \hfill \\ \end{gathered} $$

where TP stands for the number of true positive; TN stands for the number of true negative; FP stands for the number of false positive; FN stands for the number of false negative.

**Table 2:** Performance of this work vs. other object detectors

	Accuracy (%)	Precision (%)	Recall (%)
Dataset I			
This work	95.6	95	96
Fast R-CNN	91.2	92	90
R-CNN	88.5	87	90
Dataset II			
This work	94.5	94	95
Fast R-CNN	90.5	91	90
R-CNN	86.5	86	86

**Table 3:** Performance of the proposed approach vs. other methodologies, averaging dataset I and II

Method	Accuracy (%)
This work with Dataset I	95.6
This work with Dataset II	94.5
Sener et al. [28]	93.3
Rinkal et al. [29]	92.8
Yadav et at [30]	91

Experiments were carried on a computer with Intel® Core TM I3-6006U CPU @ 2 GHz. MATLAB2020a was adopted with its built-in applications such as Ground Truth Labeler, Neural Network Designer. Jetson nano was used as an embedded system test platform in Sect. 6.

### Real-time measurement of this work vs other object detectors

**Fig. 9:**
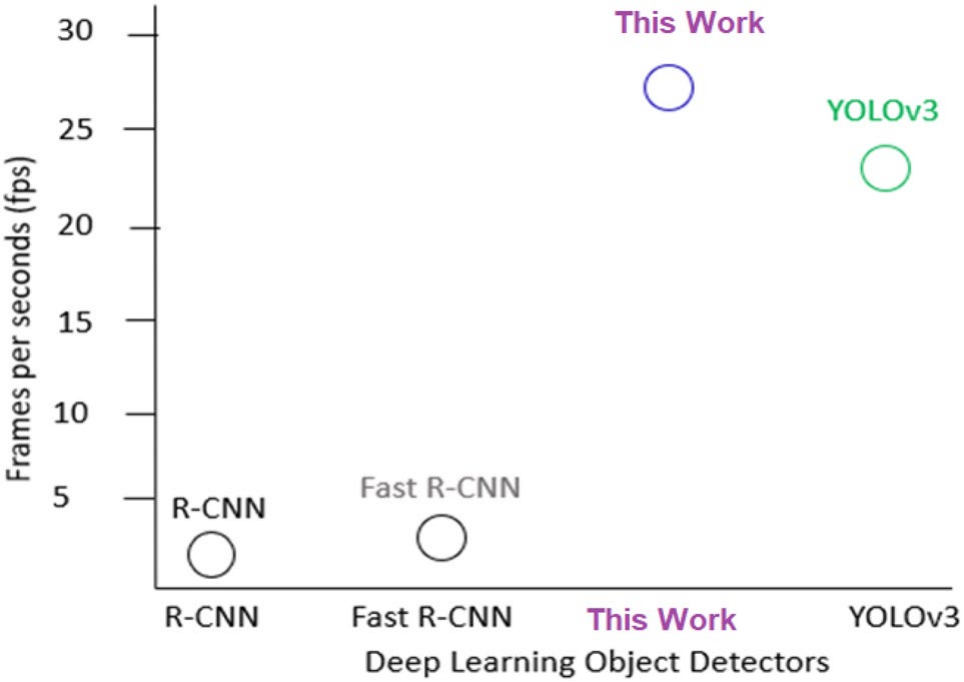
The comparison of this work vs other competing deep learning detectors (R-CNN, Fast R-CNN, and YOLOv3) for real-time detection

The main objective of this research is to detect and recognize individuals in real-time. We have to monitor and track people’s movements by utilizing a video camera. The invention and evolution of deep learning have improved the traditional ways of object detection and recognition systems. This technology is applied in several applications to identify and locate the objects in images, and it showed encouraging results for real-time detection [31]. To understand further, experiments were carried to compare the proposed approach and other deep learning detectors such as R-CNN and Fast R-CNN. MATLAB was used with our test bench of videos that were captured from a thermal camera. The three models run simultaneously while frames per second were calculating for each model. Based on results from this experiment, the neural network proposed in this work runs faster than the other two detectors (Fast R-CNN and R-CNN). Note that this work showed better results for real-time detection comparable to the method [32], which proposed YOLOv3 detector. It is observed that R-CNN and Fast R-CNN have low frames per second, which make them not suitable for real-time applications. Figure 9 shows the comparative real-time detection of this work versus other deep learning object detectors.

## Implementation of the proposed approach on embedded hardware

### Jetson nano (NVIDIA device)

Jetson nano NVIDIA system is a low-cost embedded device but a powerful computer. It costs approximately $100 [33]. Jetson nano can run various advanced neural networks including a full version of most popular deep learning (DL) and machine learning (ML) frameworks such as Pytorch, Caffe, Keras, and TensorFlow. This embedded device uses TensorRT accelerator libraries which include Jetpack packages. Jetson nano is suitable for real-time applications in different scenarios and is capable to process multiple high-definition video streams.

Jetson nano includes CPU QUAD-core ARM A57 at 1.43 GHz and GPU 128-core Maxwell. The memory of the device is 4 GB, 64-bit, LPDDR4 25.6 GB/s. Jetson nano has a USB 2.0 Micro-B, 4 × USB 3.0. A standard camera module with 8 M pixel resolution has been used in our experiments. The camera was connected to the camera serial interface (CSI) in Jetson nano. The trained neural network model and social distancing classification algorithm defined in Sect. 4 has been deployed in Jetson nano and it runs as a standalone application.

MATLAB environments and third-party packages were utilized to generate the C code of the proposed approach in the NVIDIA device. GPU coder was used for converting MATLAB code into an optimized CUDA code. Compute unified device architecture or CUDA is an extension of C programming language which is designed for NVIDIA frameworks. Jetson nano was connected to the host computer using an ethernet cable. MATLAB coder was utilized to generate C code to Jetson nano. We used a parallel computing toolbox to solve complex computational and data processing using a multicore processor and GPU. A deep learning toolbox was utilized to provide a framework to implement the neural network and algorithms in Jetson nano. GPU support package for NVIDIA is used to deploy the proposed algorithms in Jetson nano. This support package application enables the communication remotely to the targeted NVIDIA hardware. Embedded coder was used for code generation on Jetson nano. This tool improves the code generation on hardware effectively. A JetPack developer AI tool was installed in the NIVIDIA device. It is an environment variable application which is to be applicable for code generation of the proposed deep learning architecture in Jetson nano. Microsoft visual studio 2019 was installed as a compiler generate GPU code in Jetson nano. CUDA Deep Neural Network libraries were used to accelerate primitives for neural network architecture.

### Test the proposed algorithm on Jetson nano

The proposed algorithm was deployed in Jetson nano and run as a standalone application to evaluate its performance. A Raspberry Pi camera model V2 was exposed to another personal computer that simulated a number of videos that were captured from a thermal camera. While the proposed algorithm was running in the NIVIDIA device, we recorded various parameters. We measured the average frames per second for the proposed approach on Jetson nano and we compared the achived results with other different methods, see Table 4. According to the results from this experiment, our approach showed the best result for the real-time which reached up to 27 fps.

**Table 4:** The real-time measurement for the proposed approach vs other methods

Method		Real-time in (*fps*)
The proposed approach		27
Rezaei e al. [34]		24.1
Punn, et al. (YOLOv3) [32]		23
Pouw et al. [35]		10

We measured also the power consumption for Jetson nano. We removed all Jetson nano accessories such as a mouse, monitor, and keyboard. We measured the power consumption at 1.24 W when the deployed algorithm is off. When the distance classification algorithm was executed, the power consumption was recorded at 4.4 W. Table 5 shows the power measurement of the NVIDIA device in different scenarios.

**Table 5:** Power consumption measurement in different scenarios

	Algorithm status	Power measurement (W)
Jetson nano without monitor, keyboard, mouse	Off	1.24
	Running	4.40
Jetson nano with monitor, keyboard, mouse	Off	2.24
	Running	5.40

**Fig. 10:**
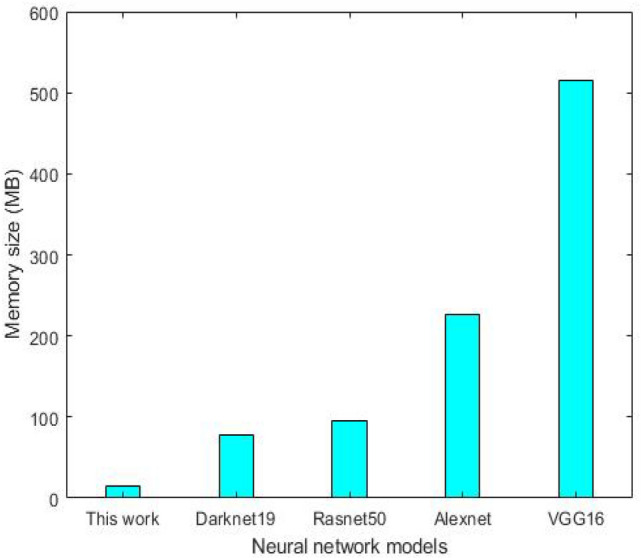
Comparison of the proposed approach verses other pre-trained models in terms of memory size

We recorded the measurement of Graphics Processing Unit (GPU) and the Central Processing Unit (CPU) % resource utilization in Jetson device. The GPU is designed to process the graphic operations and the CPU runs the operating system and applications. These characteristics are essential to assess its computation processing. The table shows the measured values for the GPU and CPU processors while the proposed algorithm was executed in the targeted hardware. Moreover, we measured the temperature of Jetson nano while the proposed approach was in execution. The temperature was measured at 54.5 °C for GPU and 54.1 for CPU, see Table 6. Further to our experiments, we measured the memory size for the deployed algorithm in Jetson nano, which is 14 MB. This is the advantage of the proposed approach in comparison to the other pre-trained models such as VGG16, Alexnet, and Resnet50 in method [29]. Figure 10 shows the comparison between the proposed approach with other pre-trained models in terms of memory size. These architectures use massive CNN layers which need a large disk size for the deployment on the targeted embedded system. This could affect real-time performance while the algorithm is running on low-cost embedded devices. This is the advantage of the proposed approach versus the pre-trained CNN models and it can be superior for real-time detection.

**Table 6:** The % resource utilization and temperature measurement for the GPU and CPU in Jetson nano while our method is running on it

	Performance %	Temperature (°C)
Jetson nano (GPU)	99	54.5
Jetson nano (CPU)	70.1	54.1

### Distributed surveillance video system for social distancing

Video surveillance cameras are an effective monitoring system for authorities to visualize how people are acting and their compliance with social distancing. In our research, we implemented a distributed surveillance camera system based on embedded devices.

**Fig. 11:**
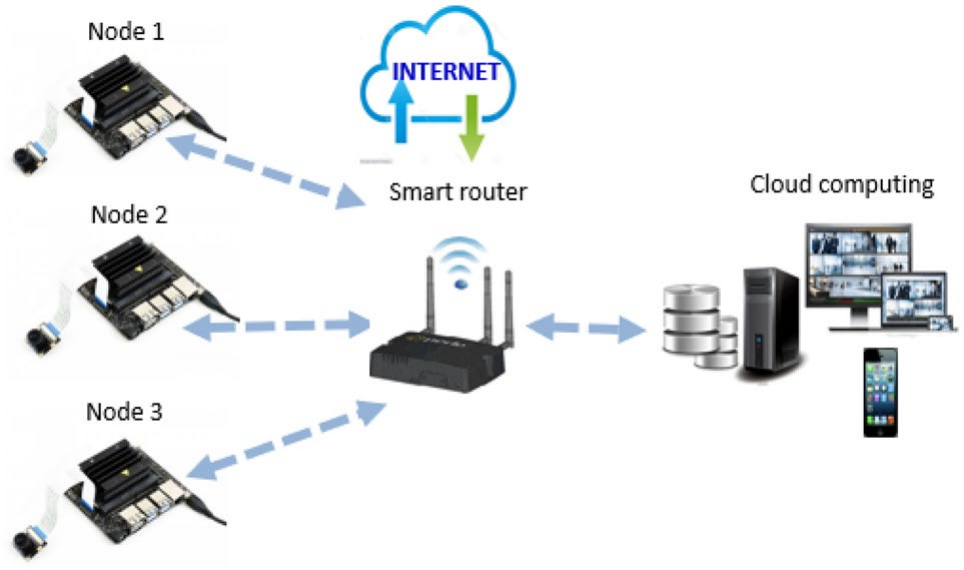
Smart Surveillance distributed video system for people detection and social distancing classification

The proposed system is composed of multiple Jetson nanodevices with, each combined with a video camera. Each camera is connected to one Jetson nanodevice, which represents a smart node in the system architecture. Jetson nanodevices were upgraded with Wi-Fi for internet connection. All Jetson nanodevices were connected to the computer through a router using a static IP address from each node. The router directs the video streaming of each node and serves as a networking device to the centralized surveillance management system (personal computer), see Fig. 11.

MobaXterm application in Windows 10 was used to establish communication between the centralized surveillance management system (personal computer) and Nvidia Jetson nano nodes [36]. The communication was established using an OpenSSH application with respect to the defined *IP address* of each node. Secure Shell or OpenSSH is a remote information communication technology protocol that allows users to control and transfer data between computers. The system is built with a multi-access point of *IP addresses* through OpenSSH sessions in the MobaXterm software*.* Each OpenSSH session communicates with Jetson nano node through its defined *IP address*. The latency time was measured between the computer and Jetson boards at 0.3 ms. The proposed approach is suitable for a distributed surveillance system that can visualize people detection and social distancing classification on thermal images from several Jetson nanodevices in one centralized surveillance management system.

## Conclusion and future work

This research presented an intelligent surveillance system for people tracking and social distancing classification based on thermal images. The proposed technique achieved promising results for people detection in terms of evaluation the accuracy and precision of the detector comparable to the other deep learning models. A specific algorithm was implemented on bounding boxes to distinguish between safe and unsafe conditions, respectively, marking as green and red the bounding box for detected persons. The proposed technique showed better results for real-time performance vs other object detectors. The proposed approach can be implemented in a distributed video surveillance system; indeed, it is a suitable solution for the authorities to visualize the compliance of people with social distancing and at the same time screening their body temperature. In the future, we will utilize this methodology on mobile cameras, e.g., mounted on an autonomous drone system, and hence drones are simpler to operate and more effective to capture fast actions of the detected objects from different angles. We will extend our research to use and experiment people detection by also applying 3-D dimensions to have three parameters (*x*, *y*, *z*), in which we can perceive uniform distribution distance in the entire image and eliminating the perspective effect. In addition to that, the newly released YOLOv4 detector [37] will be also considered.
